# Effects of depth of straw returning on maize yield potential and greenhouse gas emissions

**DOI:** 10.3389/fpls.2024.1344647

**Published:** 2024-02-21

**Authors:** Junqiang Wang, Yehui Han, Chao Zhou, Ting Xu, Zhongcheng Qu, Bo Ma, Ming Yuan, Lianxia Wang, Yang Liu, Qingchao Li, Xinying Ding, Chunrong Qian, Baoxin Ma

**Affiliations:** ^1^ Heilongjiang Academy of Agricultural Sciences, Qiqihar, China; ^2^ Animal Husbandry and Veterinary Branch of Heilongjiang Academy of Agricultural Sciences, Qiqihar, China; ^3^ Institute of Tillage and Cultivation, Heilongjiang Academy of Agricultural Sciences, Harbin, China

**Keywords:** straw returning, maize, yield potential, greenhouse gases, soil organic carbon

## Abstract

Appropriate straw incorporation has ample agronomic and environmental benefits, but most studies are limited to straw mulching or application on the soil surface. To determine the effect of depth of straw incorporation on the crop yield, soil organic carbon (SOC), total nitrogen (TN) and greenhouse gas emission, a total of 4 treatments were set up in this study, which comprised no straw returning (CK), straw returning at 15 cm (S15), straw returning at 25 cm (S25) and straw returning at 40 cm (S40). The results showed that straw incorporation significantly increased SOC, TN and C:N ratio. Compared with CK treatments, substantial increases in the grain yield (by 4.17~5.49% for S15 and 6.64~10.06% for S25) were observed under S15 and S25 treatments. S15 and S25 could significantly improve the carbon and nitrogen status of the 0-40 cm soil layer, thereby increased maize yield. The results showed that the maize yield was closely related to the soil carbon and nitrogen index of the 0-40 cm soil layer. In order to further evaluate the environmental benefits of straw returning, this study measured the global warming potential (GWP) and greenhouse gas emission intensity (GHGI). Compared with CK treatments, the GWP of S15, S25 and S40 treatments was increased by 9.35~20.37%, 4.27~7.67% and 0.72~6.14%, respectively, among which the S15 treatment contributed the most to the GWP of farmland. GHGI is an evaluation index of low-carbon agriculture at this stage, which takes into account both crop yield and global warming potential. In this study, GHGI showed a different trend from GWP. Compared with CK treatments, the S25 treatments had no significant difference in 2020, and decreased significantly in 2021 and 2022. This is due to the combined effect of maize yield and cumulative greenhouse gas emissions, indicating that the appropriate straw returning method can not only reduce the intensity of greenhouse gas emissions but also improve soil productivity and enhance the carbon sequestration effect of farmland soil, which is an ideal soil improvement and fertilization measure.

## 1 Introduction

In recent years, the impact of climate warming on natural economy and human life has become a global problem (Linquist et al., 2012). At present, it is generally believed that the increasing concentration of greenhouse gases (CO_2_, CH_4_, N_2_O) in the atmosphere was the main cause of climate warming. Among them, 10%-20% of the total anthropogenic greenhouse gas emissions had generated by agricultural activities (Smith et al., 2007). The emission of greenhouse gases from farmland comes from the direct emission of farmland soil and the indirect emission of agricultural management measures, such as tillage, irrigation, straw returning, fertilization, etc. (Baggs et al., 2003; Toma and Hatano, 2007; Saggar, 2010; Trost et al., 2013). Therefore, agricultural production is considered to be an important source of greenhouse gas emissions.

As a carrier of material, energy and nutrients, straw is a valuable renewable natural resources (Amaya et al., 2007). China is a large agricultural country, which had produced a huge amount of crop straw every year, more than 800 million tons (Xia et al., 2014; Liu et al., 2021; Zhong et al., 2022). The content of N, P, K and other nutrient elements in straw was rich. As an organic fertilizer resource, it can be equivalent to 40% of the amount of chemical fertilizer used in China (Zhuang et al., 2020). The traditional treatments of incineration will not only cause serious environmental pollution, but also a great waste of resources. In recent years, China ‘s farmland farming model has changed significantly. The crop straw is no longer used as fuel, and the common agricultural practice was returned the straw to the field, which not only improves soil fertility but also reduces air pollution caused by crop straw burning (Gao et al., 2011). Straw returning can make the carbon in the straw return to the soil to participate in the carbon cycle, which can not only reduced the carbon output of the farmland ecosystem but also increased the soil organic matter content and improve the soil fertility, so as to realize the reuse of agricultural resources (Mu et al., 2016; Liang et al., 2017; Adimassu et al., 2019; Smitha et al., 2019). Some studies have shown that straw returning can stimulate the microorganisms in the soil to produce a priming effect (Kuzyakov et al., 2000), increase microbial activity, accelerate the decomposition rate of soil organic matter, and thus affect the production and emission of soil greenhouse gases. However, the current research results on the increase or decrease of greenhouse gas emissions caused by straw returning are still uncertain.

Northeast China is the main grain producing area in China. In recent years, with the increase of population growth and the improvement of living standards, higher requirements have been put forward for food production, environmental friendliness and sustainable development. People have made fruitful explorations in many fields such as high-yield cultivation, breeding and biotechnology. However, with the increase of crop yield, the biomass of straw has also increased significantly (Tian et al., 2020). In the past many years, due to the long-term shallow tillage of small agricultural machinery and the predatory production mode of large-scale application of chemical fertilizers, the comprehensive production capacity of farmland soil in Northeast China has declined sharply (Tian et al., 2019; Sui et al., 2020). Although the crop straw is the main source of organic materials for soil fertilization, straw burning is the most common straw treatment method, which was not only a waste of resources, but also caused serious environmental pollution. Therefore, aiming at the straw problem existing in the production of spring maize in Northeast China. A total of 4 treatments were set up in this study, which were no straw returning (CK), straw returning at 15 cm (S15), straw returning at 25 cm (S25) and straw returning at 40 cm (S40). By analyzing the effects of straw returning on the maize yield, physical and chemical properties of farmland soil and CO_2_ and N_2_O emissions, global warming potential (GWP) and greenhouse gas emission intensity (GHGI) were measured, and the regulation effect of straw returning depth on rice production potential and greenhouse gas emission reduction in paddy field was comprehensively evaluated to determine the optimal straw returning depth. It is expected that the research results will be of great significance to the scientific and rational use of straw and greenhouse gas emission reduction.

## 2 Materials and methods

### 2.1 Site description

The experiment was conducted in the Qiqihar maize experimental station of the Heilongjiang Academy of Agricultural Sciences, which is located in Qiqihar, Heilongjiang Province, China (46°52′N, 123°46′E) during the maize growing season (May to October) from 2020 to 2022. The test area belongs to the mid-temperate continental monsoon climate, which is characterized by dry and windy spring and warm and rainy summer. The annual precipitation is 477 mm, and a frost-free period of approximately 130 days. The soil type was Argosols (FAO classification) and the basic key properties are shown in **Table 1**.

**Table 1:** The physicochemical property of composite topsoil samples (0-60 cm).

Soil layer	Organic matter	Total N content	Rapidly available N	Rapidly available P	Rapidly available K	Value of PH
	(g kg^-1^)	(g kg^-1^)	(mg kg^-1)^	(mg kg^-1)^	(mg kg^-1)^	
0-20cm	19.12	0.75	67.52	23.21	146.8	7.23
20-40cm	18.37	0.54	64.74	22.36	140.6	7.27
40-60cm	17.37	0.53	61.74	20.36	137.6	7.25

### 2.2 Experimental materials and design

The experiment began in May 2020 and ended in October 2022. The test crop was maize and the variety was Nendan 29. The experiment comprised of four treatments as follows: no straw incorporation (CK), straw incorporation at 15 cm soil depth (S15), straw incorporation at 25 cm (S25) and straw incorporation at 40 cm (S40). Straw returning rate was 8000 kg hm^-1^. The treatments were arranged into a randomized block design and replicated three times. Nitrogen rates were 180 kg ha^-1^, and nitrogen fertilizer was applied according to the different stages, with base fertilizer and top-dressing fertilizer following a proportion of 1:2. Phosphorus (P_2_O_5_) rates were 90 kg ha^-1^ and potassium (K_2_O) rates were 120 kg ha^-1^, and phosphoru and potassium were applied as base fertilizer at one time. N, P, and K fertilizers was used urea, Ca(H_2_PO_4_)_2_ and K_2_SO_4_, respectively. Other management measures were consistent with local agronomic practices including weeding and spraying insecticides throughout the experiment.

### 2.3 Sampling and measurement

#### 2.3.1 Grain yield

Yield samples of maize were collected randomly from 1 m double rows per plot at maturity. Grain yield was standardized to a moisture content of 0.14 g H_2_O g^−1^.

#### 2.3.2 Determination of soil carbon and nitrogen content

After maize harvest, soil samples were collected in three layers (0-20 cm, 20-40 cm, and 40-60 cm) using a soil drill with a diameter of 3 cm. Five points were randomly selected from each micro-area, and the soil of the same soil layer was uniformly mixed as a sample. The soil samples were placed in a cool and ventilated place, dried and ground through a 0.15 mm sieve to determine soil organic carbon (SOC) and soil total nitrogen (TN) content. The SOC was determined by potassium dichromate external heating method (Lu, 2000), and the TN was determined by Kjeldahl apparatus (Kjeltec8400, FOSS, Denmark). The SOC and TN stock was calculated using the equal weight method (Ellert and Bettany, 1995; Xue et al., 2015), to eliminate the bias in the calculation of SOC and TN stocks caused by different plough layer thickness due to tillage. The ratio of SOC to TN was defined as soil carbon-nitrogen (C:N) ratio (Blanco-Canqui and Lal, 2008).


$$\text{Soil organic carbon stocks }\left(\text{SOC stocks},{\text{ Mg ha}}^{-1}\right)=\text{SOC}\times \text{BD}\times \text{soil depth}\times 100$$

$$\text{Soil total nitrogen stocks }\left(\text{STN stocks},{\text{ Mg ha}}^{-1}\right)=\text{TN}\times \text{BD}\times \text{soil depth}\times 100$$

#### 2.3.3 Measurement of greenhouse gas emission fluxes

The emission fluxes of soil greenhouse gases CO_2_ and N_2_O were measured by static chamber method. Sampling once every 7 days during the growth period and once every 2 days after fertilization. Each treatment was placed in three static observation boxes, which were placed between two rows of corn. The sampling time was from 9:00 to 10:00 in the morning. The gas was collected every 10 min for a total of 5 times, and 30 mL of gas was collected in the trachea each time. Immediately after the sample collection was completed, the sample was taken back to the laboratory and analyzed within 24 hours using a gas chromatograph equipped with an ECD (Electron Capture Detector) and a FID (Flame Ionization Detector) detector (Agilent 7890A, Shanghai, China).The formula of CO_2_ and N_2_O emission flux was as follows:


F=ρ×H×ΔC/Δt×273/(273+T)

F is CO_2_ emission flux or N_2_O emission flux; ρ is the density of CO_2_ or N_2_O in the standard state; H is the height of the closed box (m); ΔC/Δt is the change rate of CO_2_ or N_2_O concentration in the test chamber; T is the average temperature (°C) in the chamber during the sampling process.

The formula of cumulative CO_2_ or N_2_O emissions during the growing season was as follows:


$$CE=\sum \left\{\frac{F_{i}+F_{\left(i+1\right)}}{2}\times 10^{-3}\times d\times 24\times 10\right\}$$

CE is the cumulative emission of gas (CO_2_ or N_2_O), F_i_ and F_(i+1)_ is the gas emission fluxes (mg m^-2^ h^-1^) in two consecutive adjacent sampling periods, and d is the number of days between two consecutive adjacent sampling periods.

On a 100-year timescale, the warming potential of N_2_O is 298. The formula of global warming potential (GWP) was as follows:


$$GWP=CE_{CO_{2}}+\left(CE_{N_{2}O}\times 298\right)$$

Greenhouse gas intensity (GHGI) is an index for comprehensive evaluation of greenhouse effect. The formula was as follows:


$$GHGI=\frac{GWP}{Grain\;yield}$$

### 2.4 Statistical analysis

Data analyzes were performed using Excel 2019 and SPSS 23.0 software. Significant differences between treatments were indicated by different letters at p< 0.05 level according to Fisher’s LSD. Graphs were drawn with Origin 2018 software (OriginLab, Northampton, MA, USA), R software (Available online: http://www.r-project.org/) and Adobe Illustrator CS6 (Adobe Systems Inc., CA, USA).

## 3 Results

### 3.1 Grain yield

**Figure 1:**
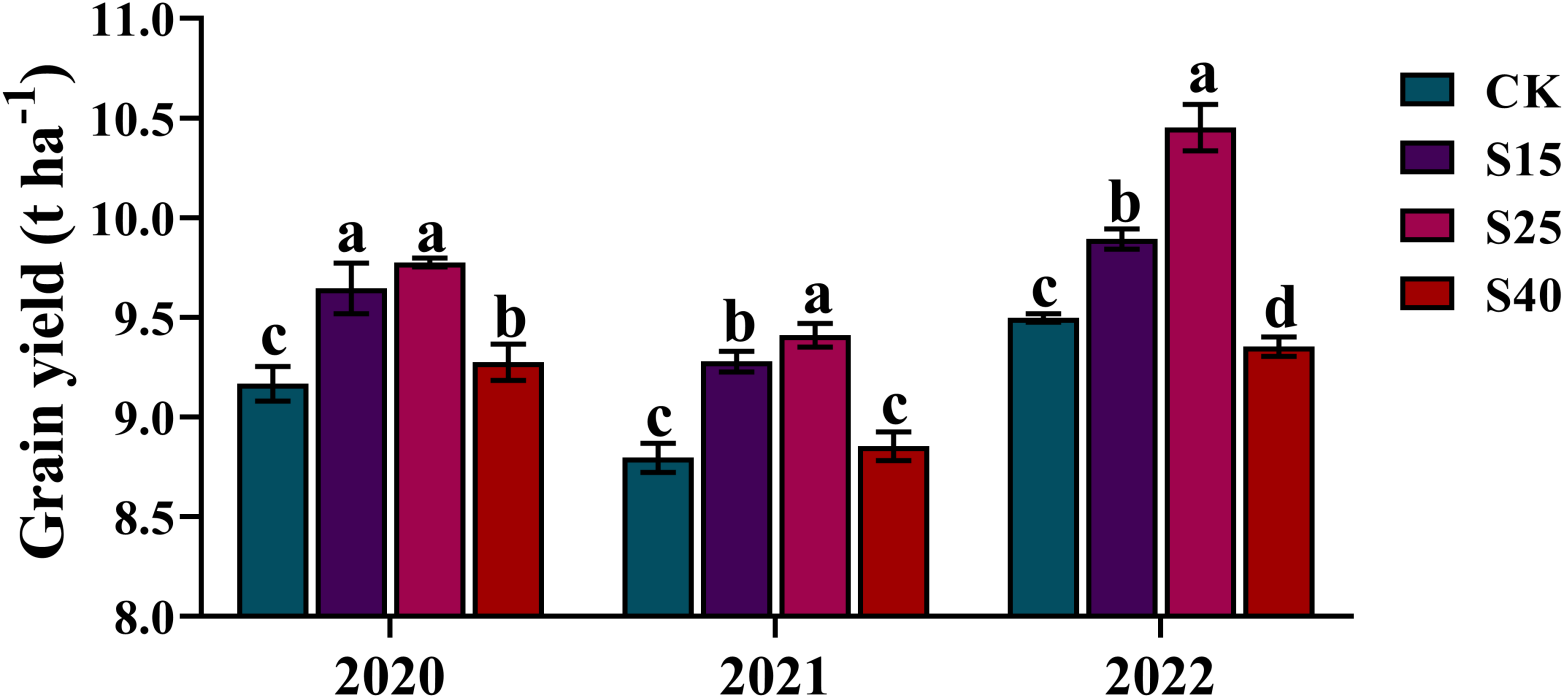
Effects of depth of straw returning on maize yield. For each year, bars followed by the different letters are significantly different at P< 0.05. CK: no straw returning; S15: straw returning at 15 cm soil depth; S25: straw returning at 25 cm; S40: straw returning at 40 cm.

As shown in **Figure 1**, depth of straw returning significantly affected the maize yield. Compared with CK treatments, S15 and S25 treatments were significantly increased the maize yield, and was the highest under S25 treatments. In 2020, the maize yield increased significantly by 6.64% under S25 treatments and 5.22% under S15 treatments, respectively. In 2021, the maize yield increased significantly by 6.99% under S25 treatments and 5.49% under S15 treatments, respectively. In 2022, the maize yield increased significantly by 10.06% under S25 treatments and 4.17% under S15 treatments, respectively. While the S40 treatments had little effect on the maize yield, the maize yield increased significantly by 1.18% in 2020 and decreased by 1.51% in 2022.

### 3.2 Soil organic carbon and SOC stocks

The depth distribution of SOC and SOC stocks was significantly affected by depth of straw returning (**Table 2**). Compared with the CK treatments, at the 0-20 cm depth, the SOC was the largest under S15 treatments, which increased by 8.50~14.22%, and the SOC stock was the largest under S25 treatments, which increased by 9.71~22.34%. Compared with the CK treatments, at the 20-40 cm depth, the SOC was the largest under S25 treatments, which increased by 9.91~22.55%, and the SOC stock was the largest under S40 treatments, which increased by 4.15~16.61%. Compared with the CK treatment, at the 40-60 cm depth, the SOC was the largest under S40 treatments, which increased by 7.93~18.60%, and the SOC stock was the largest under S40 treatments, which increased by 4.88~17.30%.

**Table 2:** Depth distribution of SOC (g kg^-1^) and SOC stocks under different straw returning treatments.

Soil layer	Treatments	2020		2021		2022	
		SOC content	SOC stocks	SOC content	SOC stocks	SOC content	SOC stocks
0-20 cm	CK	8.12c	18.93c	8.13d	18.88d	8.09d	18.60c
	S15	8.81a	19.65b	9.02a	20.71b	9.24a	20.84b
	S25	8.63b	20.81a	8.74b	21.85a	8.96b	22.80a
	S40	8.42b	19.40b	8.43c	20.38c	8.42c	20.31b
20-40 cm	CK	6.86c	15.87b	6.79c	15.51c	6.74c	15.07c
	S15	7.12b	16.24a	7.45b	16.50b	7.55b	16.67b
	S25	7.54a	16.30a	7.86a	17.21a	8.26a	17.47a
	S40	7.03b	14.43a	7.33b	17.66a	7.36b	17.58a
40-60 cm	CK	5.55b	16.43c	5.46c	16.05c	5.27c	15.49c
	S15	5.68a	16.81b	5.81b	17.08b	5.83b	17.14b
	S25	5.70a	16.87b	6.06a	17.82a	6.11a	17.96a
	S40	5.99a	17.23a	6.22a	18.29a	6.25a	18.18a
0-40 cm	CK	7.49b	34.81c	7.46c	34.38d	7.42c	33.67c
(average)	S15	7.97a	35.90b	8.24a	37.21c	8.40a	37.51b
	S25	8.09a	37.11a	8.30a	39.06a	8.61a	40.27a
	S40	7.73b	36.53a	7.88b	38.04b	7.89b	38.19b
0-60 cm	CK	6.84b	51.23c	6.79b	50.44c	6.70b	49.17c
(average)	S15	7.20a	52.71b	7.43a	54.29b	7.54a	54.65b
	S25	7.29a	53.98a	7.55a	56.88a	7.78a	58.24a
	S40	7.15a	54.26a	7.33a	56.33a	7.34a	56.56a

Different small letters represent significant differences among treatments

### 3.3 Total nitrogen and STN stocks

The depth distribution of TN and STN stocks were significantly affected by depth of straw returning (**Table 3**). Compared with the CK treatments, at the 0-20 cm depth, the TN and STN stock was the largest under S15 treatments, which increased by 3.02~10.18% and 2.15~8.32%, respectively. Compared with the CK treatments, at the 20-40 cm depth, the TN and STN stock was the largest under S25 treatments, which increased by 7.32~12.11% and 6.77~12.65%, respectively. Compared with the CK treatments, at the 40-60 cm depth, the TN and STN stock was the largest under S40 treatments, which increased by 4.17~14.88% and 2.47~14.98%, respectively.

**Table 3:** Depth distribution of TN (g kg^-1^) and STN stocks under different straw returning treatments.

Soil layer	Treatments	2020		2021		2022	
		TN content	STN stocks	TN content	STN stocks	TN content	STN stocks
0-20 cm	CK	0.716c	0.711b	0.704b	1.977b	1.977c	1.944c
	S15	0.738a	0.755a	0.776a	2.020a	2.070a	2.106a
	S25	0.722b	0.741a	0.754a	1.990a	2.049a	2.070a
	S40	0.714c	0.722b	0.724b	1.965c	1.999b	1.983b
20-40 cm	CK	0.577	0.567	0.573	1.651	1.611	1.639
	S15	0.595	0.606	0.612	1.702	1.719	1.741
	S25	0.619	0.639	0.641	1.762	1.806	1.827
	S40	0.604	0.613	0.622	1.724	1.739	1.777
40-60 cm	CK	0.569	0.554	0.534	1.685	1.628	1.569
	S15	0.564	0.55	0.545	1.669	1.616	1.603
	S25	0.562	0.591	0.607	1.665	1.736	1.785
	S40	0.593	0.616	0.613	1.727	1.754	1.804
0-40 cm	CK	0.647	0.639	0.639	3.628	3.588	3.583
(average)	S15	0.667	0.681	0.694	3.722	3.789	3.847
	S25	0.671	0.69	0.698	3.752	3.856	3.897
	S40	0.659	0.668	0.673	3.689	3.737	3.76
0-60 cm	CK	0.621	0.611	0.604	5.313	5.217	5.152
(average)	S15	0.632	0.637	0.644	5.391	5.405	5.45
	S25	0.635	0.657	0.668	5.417	5.592	5.682
	S40	0.634	0.644	0.653	5.415	5.491	5.564

### 3.4 Soil C:N ratio

**Figure 2:**
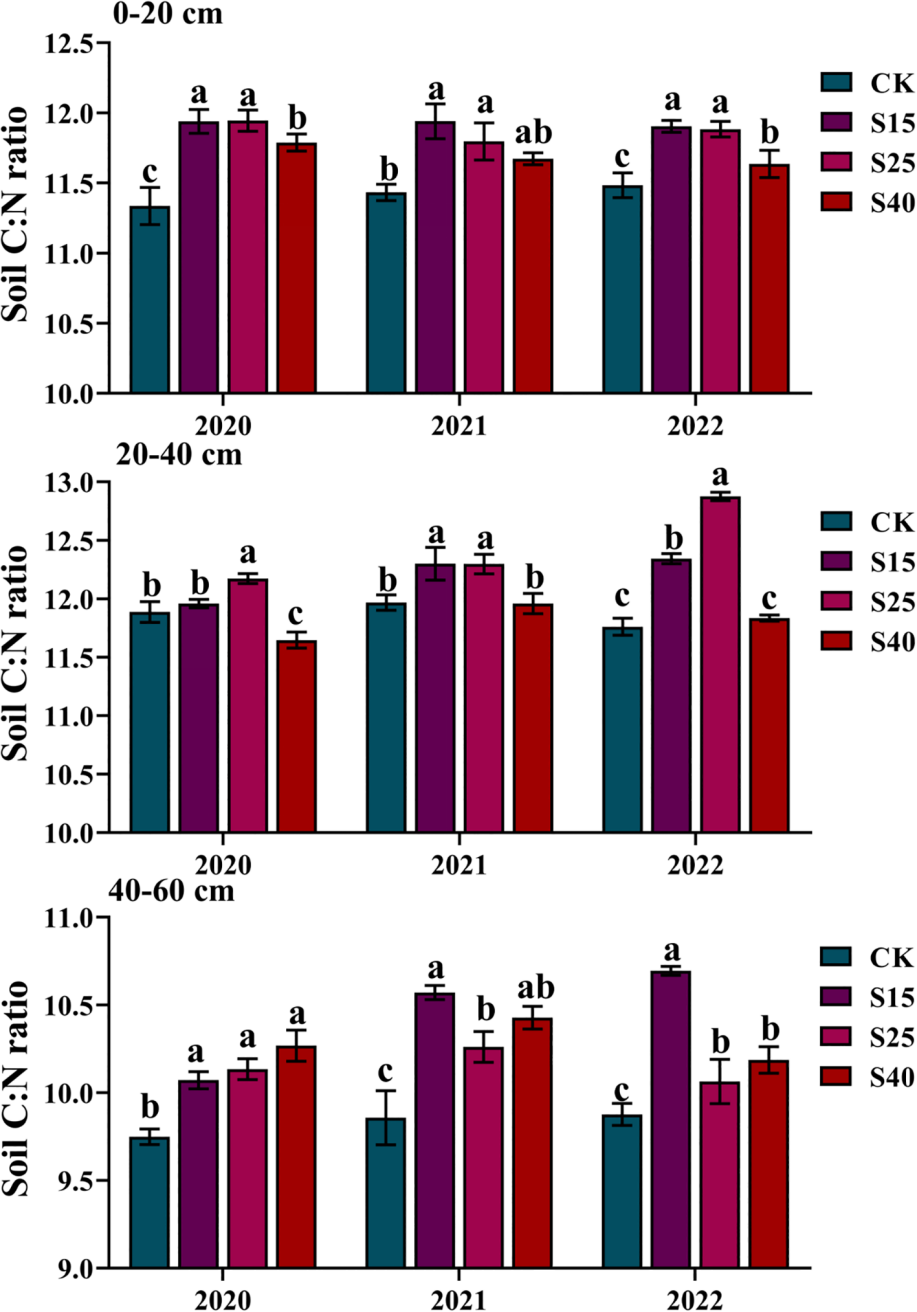
Effects of depth of straw returning on C:N ratio. For each soil layer, bars with different letters differ significantly at P< 0.05. CK, no straw returning; S15, straw returning at 15 cm soil depth; S25, straw returning at 25 cm; S40, straw returning at 40 cm.

The depth distribution of C:N ratio was significantly affected by depth of straw returning. Compared with the CK treatments, at the 0-20 cm depth, the soil C:N ratio was the largest under S15 treatments, which increased by 3.66~5.32%. Compared with the CK treatments, at the 20-40 cm depth, the soil C:N ratio was the largest under S25 treatments, which increased by 2.41~9.48%. Compared with the CK treatments, at the 40-60 cm depth, the soil C:N ratio was the largest under S40 treatment which increased by 5.32% in 2020, which was the largest under S40 treatments which increased by7.24 ~8.29% in 2021 and 2022 (**Figure 2**).

### 3.5 Relationships of grain yield versus SOC, TN and C:N ratio

**Figure 3:**
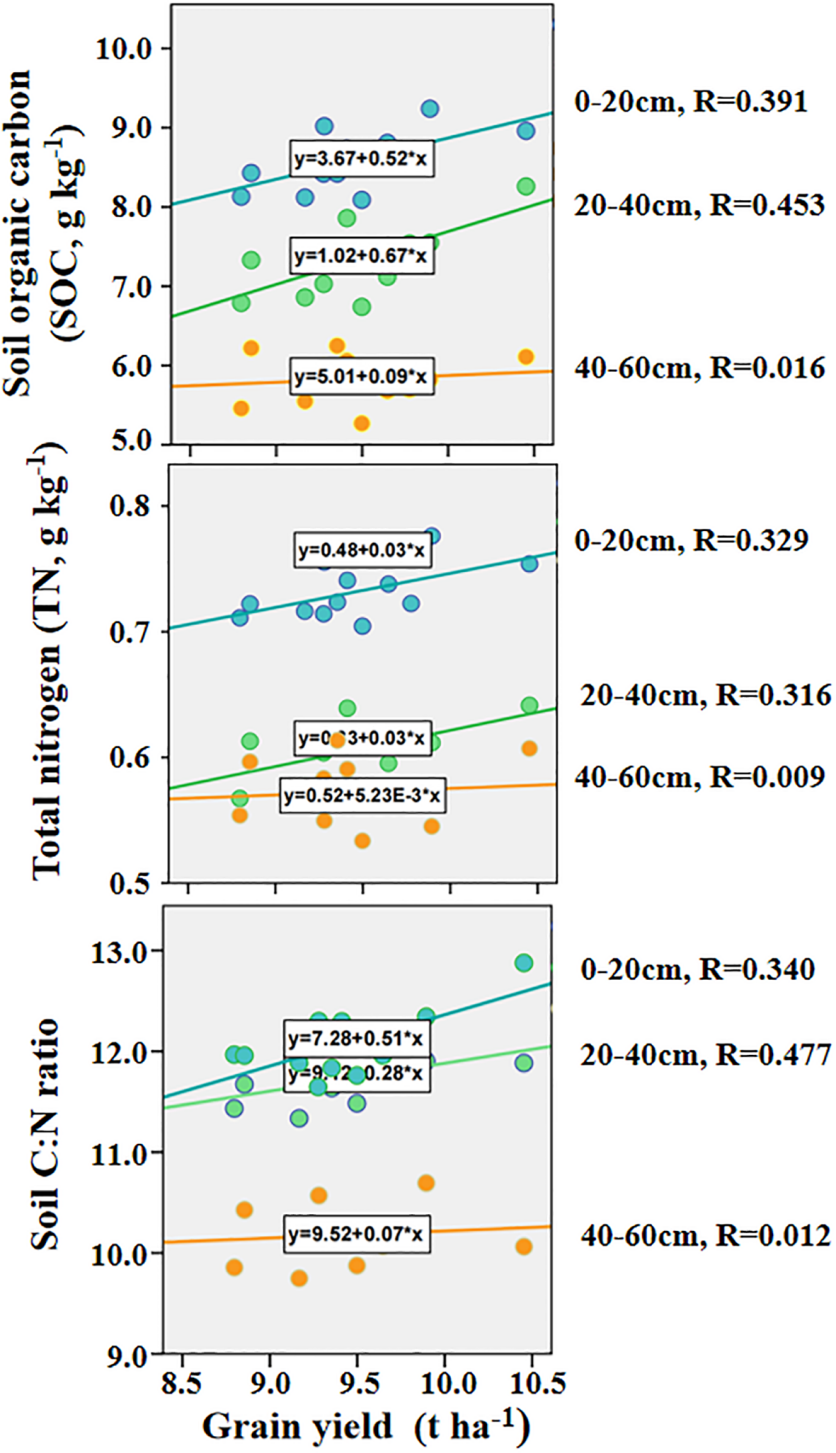
The relationship between Grain yield and SOC, TN and C:N ratio at different soil depths from 2020 to 2022. GN, grain yield, SOC, soil organic carbon, TN, total nitrogen, C:N ratio, carbon-nitrogen ratio.

Correlation analysis results also showed that the grain yield was significant related to the SOC, TN and soil C:N ratio (**Figure 3**). The grain yield had significantly positive correlations with the SOC, TN and soil C:N ratio at the 0-20 cm and 20-40 cm depth, while was not significantly correlation with the SOC, TN and soil C:N ratio at the 40-60 cm depth.

### 3.6 The feature of greenhouse gases emission

**Figure 4:**
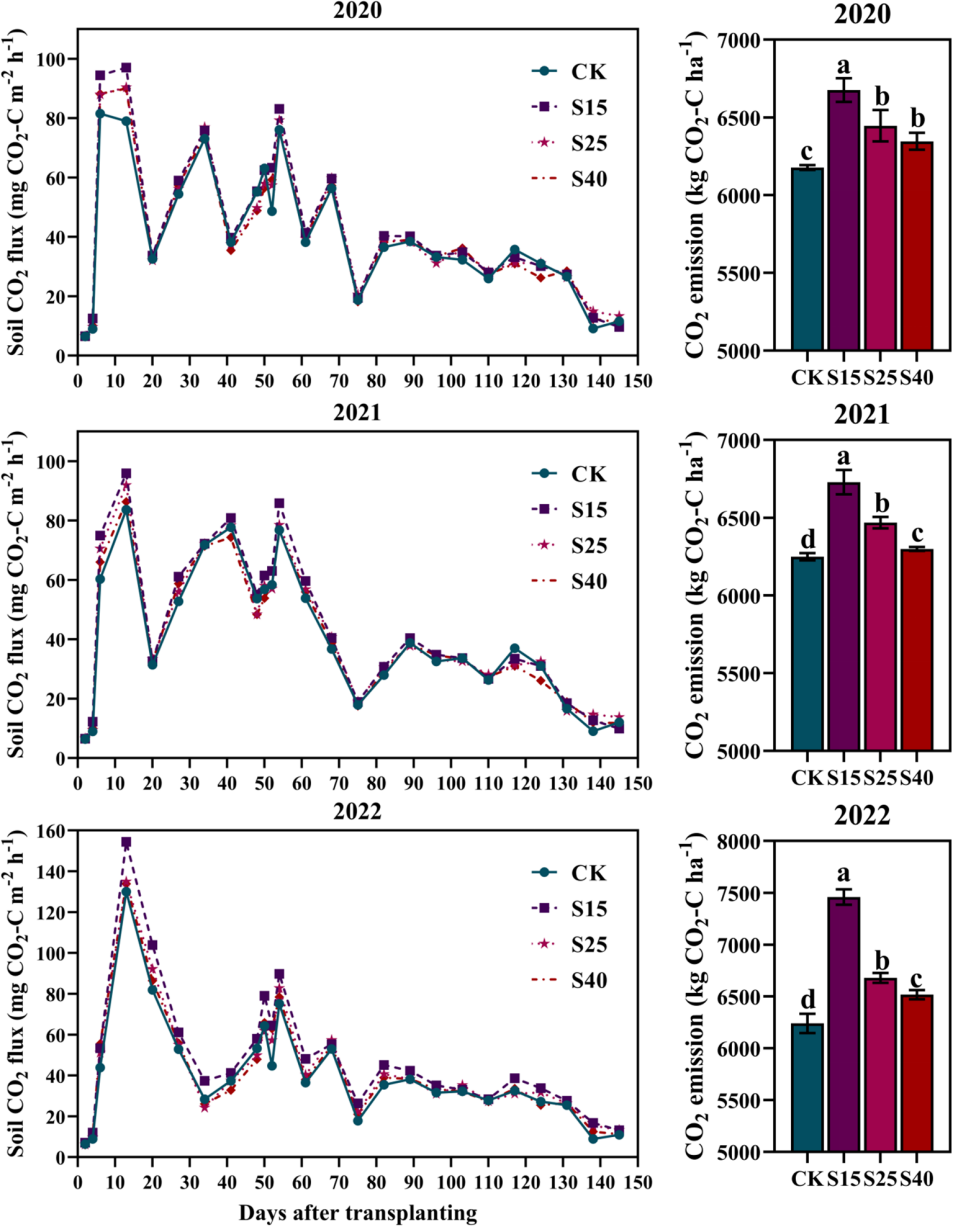
Effects of depth of straw returning on dynamics of CO_2_ fluxes and cumulative CO_2_ emissions. For each year, bars followed by the different letters are significantly different at P< 0.05. CK, no straw returning; S15, straw returning at 15 cm soil depth; S25, straw returning at 25 cm; S40, straw returning at 40 cm.

The dynamic changes of the soil CO_2_ flux and CO_2_ emission in maize growing season under all depth of straw returning treatments were shown in **Figure 4**. The soil CO_2_ flux of each treatments showed an obvious bimodal change trend during the whole maize growing season. In the early stage of maize growth, the soil CO_2_ flux was larger and then gradually decreased, and reached the peak of emission flux in the middle stage of growth, and then the emission flux decreased. As shown in **Figure 4**, depth of straw returning was significantly increased the CO_2_ emission, and was the highest under S15 treatments, which was increased by 7.67~19.54% compared with the CK treatments.

**Figure 5:**
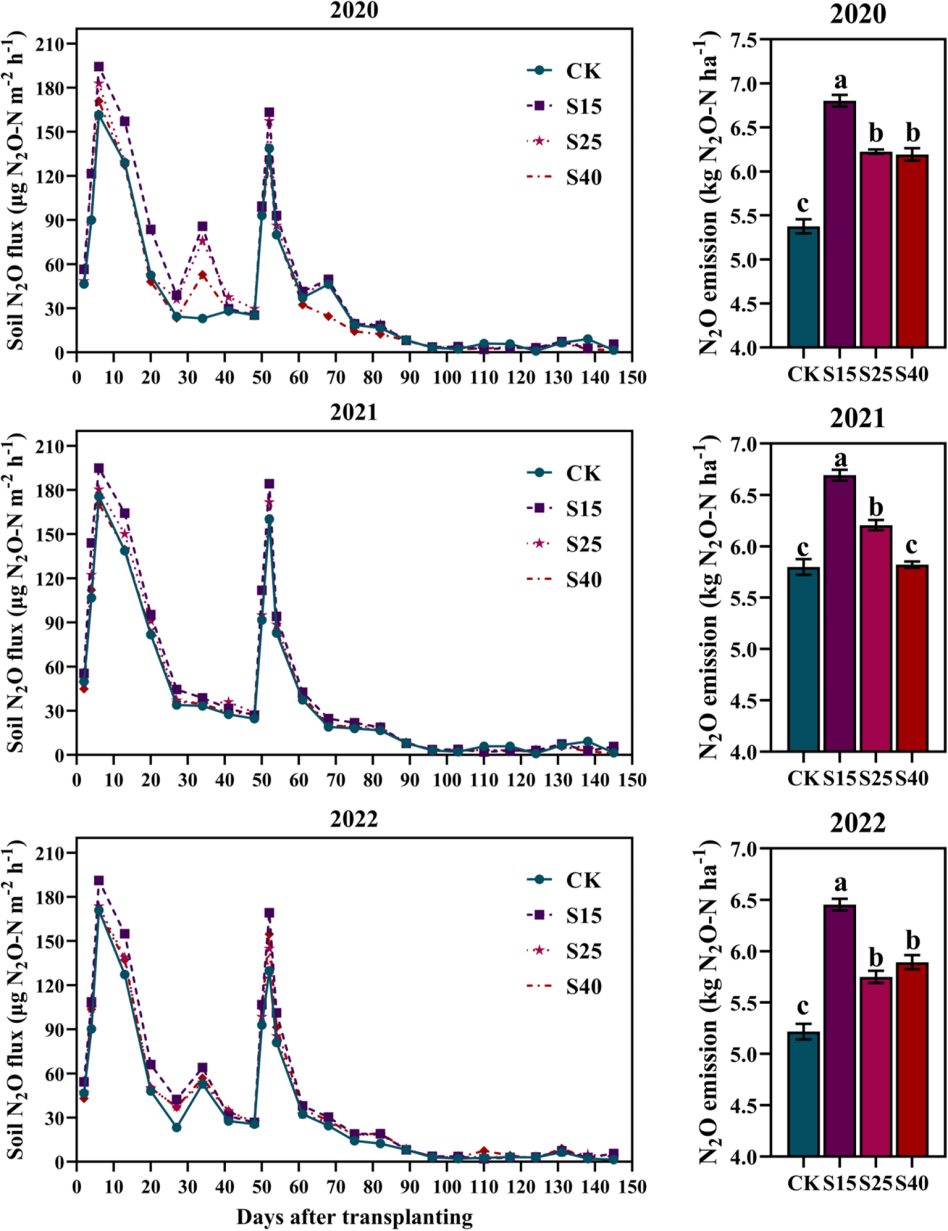
Effects of depth of straw returning on dynamics of N_2_O fluxes and cumulative N_2_O emissions. For each year, bars followed by the different letters are significantly different at P< 0.05. CK, no straw returning; S15, straw returning at 15 cm soil depth; S25, straw returning at 25 cm; S40, straw returning at 40 cm.

The dynamic changes of the soil N_2_O flux and N_2_O emission in maize growing season under all depth of straw returning treatments were shown in **Figure 5**. The soil N_2_O flux of each treatment showed an obvious bimodal change trend during the whole maize growing season. In the early stage of maize growth, the soil N_2_O flux was larger and then gradually decreased, and reached the peak of emission flux in the middle stage of growth, and then the emission flux decreased. It can be seen that the peak value of the soil N_2_O flux is roughly the same as that of fertilization period. The first peak appears after base fertilizer, and the second peak appears after top-dressing fertilizer, indicating that fertilization is the main factor affecting the soil N_2_O flux. As shown in **Figure 5**, depth of straw returning treatments was significantly increased the N_2_O emission, and was the highest under S15 treatments, which increased by 15.41~26.56% compared with the CK treatments.

### 3.7 Estimation of global warming potential and greenhouse gas emission intensity

**Figure 6:**
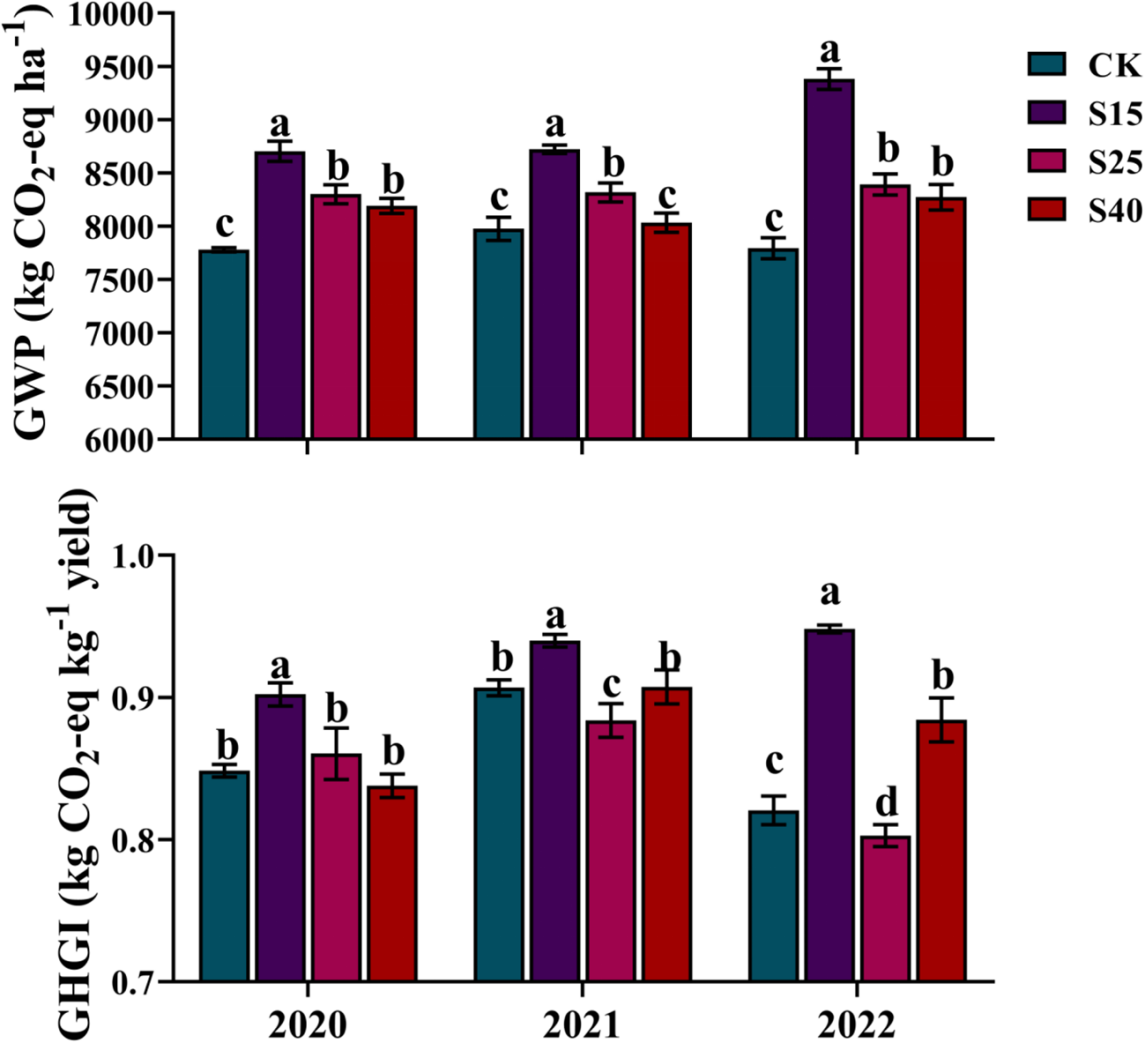
Effects of depth of straw returning on GWP and GHGI. For each year, bars followed by the different letters are significantly different at P< 0.05. CK, no straw returning; S15, straw returning at 15 cm soil depth; S25, straw returning at 25 cm; S40, straw returning at 40 cm. GWP, global warming potential, GHGI, greenhouse gas intensity.

In the maize growing season, the GWP mainly comes from the CO_2_ and N_2_O emissions. In this study, the estimated results of the GWP and GHGI under all depth of straw returning treatments in maize growing season were shown in **Figure 6**. Compared with CK treatments, the GWP of S15, S25 and S40 treatments was increased by 9.35~20.37%, 4.27~7.67% and 0.72~6.14%, respectively, among which S15 treatment contributed the most to the GWP of farmland. The GHGI is an evaluation index of low-carbon agriculture at this stage, which takes into account both crop yield and global warming potential. In this study, the GHGI was shown a different trend from the GWP. Compared with CK treatments, S25 treatments was no significant difference in 2020, and was decreased significantly in 2021 and 2022. This is due to the combined effect of the maize yield and cumulative greenhouse gas emissions, indicating that the appropriate straw returning method can not only reduce the intensity of greenhouse gas emissions but also improve soil productivity and enhance the carbon sequestration effect of farmland soil, which is an ideal soil improvement and fertilization measure.

## 4 Discussion

### 4.1 Effects of different straw returning depths on soil nutrients and grain yield

Different straw returning methods will affect the distribution of straw in the tillage layer, and the position of straw will affect the spatial distribution of the SOC and TN (Puget and Lal, 2005; Du et al., 2010). In this study, the SOC and TN near the straw position were higher than those without straw. Studies have also shown that the SOC and TN in the soil profile is affected by the content of straw and soil organic matter (Turmel et al., 2015). Some studies have shown that straw returning can cause deep soil disturbance and promote the mineralization of organic matter contained in the soil itself (Devêvre and Horwáth, 2000). Therefore, the carbon and nitrogen released by straw decomposition and the mineralization of soil organic matter may be the two main reasons for the influence of the SOC and TN in the different soil layers. The soil C:N ratio directly affects the carbon-nitrogen cycle, carbon-nitrogen interaction and the stability of soil organic matter in farmland ecosystem (Russell et al., 2005; Tong et al., 2009). Similar to the SOC and TN, different tillage depths had a significant effect on the soil C:N ratio, and the soil layer near the straw had a higher C:N ratio. Consistent with previous studies (Puget and Lal, 2005), this study found that depth of straw returning treatments helped to improve the soil C:N ratio. According to the analysis, the improvement effect is mainly due to the fact that the carbon release rate of straw is higher than the nitrogen release rate. The phenomenon of carbon fixation and nitrogen mineralization increase is common in depth of straw returning treatments under the environment of high carbon-nitrogen ratio, which may be mainly due to straw return treatments changed the soil carbon and nitrogen status (Kramer et al., 2013; Laird and Chang, 2013). Similar to the SOC and TN, the SOC and STN stocks were also higher in the position close to the straw returning. Compared with S40 treatments, S15 and S25 treatments significantly increased the SOC and STN stocks in the 0-40 cm layer. Previous studies have shown that this may be because under the straw returning treatments, the higher carbon and nitrogen release rate in the soil layer of straw returning promoted the significant increase of the SOC and STN content, thus increasing the upper the SOC and STN stocks.

Increasing the yield per unit area on the basis of limited cultivated land is helpful to ensure food security. Important factors affecting the crop yield include temperature, sunshine, precipitation, fertilization management and tillage pattern (Hou et al., 2012; Jat et al., 2018; Tian S. Z., et al., 2016). Improving soil nutrient status and nutrient use efficiency is of great significance to ensure high and stable yield of crops and sustainable production of farmland (Xin et al., 2019). Existing studies have shown that in most soil use types, straw returning treatments can increase the soil nitrogen content, crop nitrogen use efficiency compared with no straw returning treatments (Liang et al., 2017; Smitha et al., 2019). Under the condition of dry farmland soil environment in Northeast China, the maize yield was effectively improved under the condition of conventional shallow straw returning. In this study showed that the maize yield increased significantly with straw returning treatments, especially under S25 treatments, which is similar to some previous research results (Cai et al., 2014; Tian S. Z., et al., 2016; Zhang et al., 2017). Depth of straw returning treatments was affected soil bulk density and improved crop root architecture, thus promoted the absorption and utilization of nutrients and water to ensure the healthy growth and development of crops (Huang et al., 2013). In this study, the correlation analysis showed that the maize yield was significantly positively correlated with the STC, TN and C:N ratio in the 0-20cm and 20-40cm soil layers, and not significantly correlated with the 40-60cm soil layers. These results indicate that straw returning was beneficial to increase the fixation of the STC and TN in the plough layer, thereby increasing the maize yield. It was further explained that the SOC and TN in the 0 ~ 40 cm soil layer could be used as key parameters for maize growth (Kautz et al., 2013; Raiesi, 2017; Zhang et al., 2018).

### 4.2 Effects of different straw returning depths on greenhouse gas emissions

The ultimate goal of agricultural production is to take into account both economic and environmental benefits, and to ensure the sustainable development of agriculture while increasing the economic yield of crops. In this study, the emission of soil CO_2_ increased under all depth of straw returning treatments, which was the same conclusion as some study (Oorts et al., 2007; Bavin et al., 2009; Lenka and Lal, 2013). It shows that straw returning accelerates the decomposition of organic matter and the conversion rate of mineral nutrients by soil microorganisms, thus increased the emission of CO_2_. Depth of straw returning treatments have different effects on the environment of different soil layers, and the effects on the CO_2_ emissions were also different. In this study, the CO_2_ emissions decreased significantly with the increase of straw returning depth. Compared with S15 and S25 treatments, the CO_2_ emission flux under the S40 treatment was lower under S40 treatments. This study believes that on the one hand, when the straw were returned to the 15 cm and 25 cm soil layers, the soil temperature was higher than that of the 40 cm soil layers, which promotes the CO_2_ emissions. On the other hand, when the straw was returned to 40 cm, the deep water content of the soil layer greatly reduced the diffusion rate of CO_2_ in the soil pores, so the diffusion of CO_2_ to the ground decreased. In addition, some studies have also shown that with the increase of soil depth, soil catalase activity gradually decreased. When straw was returned to 40 cm, aerobic microorganisms increased less, respiration was relatively weak, and the CO_2_ emissions were reduced.

There are different views on the impact of straw returning on the N_2_O emissions. Some studies have suggested that straw returning has increased the N_2_O emissions by changing soil characteristics and stimulating soil microbial activity, thereby promoting denitrification (Sey et al., 2008; Xu et al., 2017; Hu et al., 2019). This study found that depth of straw returning treatments increased the soil N_2_O emissions, with significant peaks on the 6th and 52nd days depth of straw returning treatments, which may be related to fertilization. Fertilization provided a large amount of available nitrogen for soil microorganisms, accelerate nitrification, denitrification and mineralization, and thus promoted the N_2_O emissions (Qin et al., 2012; Hu et al., 2019). Soil NH_4_^+^-N and NO_3_^–^N are the direct substrates of nitrification and denitrification, and also directly affected the amount of the N_2_O emissions (Azeem et al., 2014). Therefore, the emission of N_2_O is based on the concentration of available nitrogen in the soil. Straw returning to different soil layers increased the concentration of available nitrogen and the N_2_O emissions (Karen and Keith, 2003; Horváth et al., 2010; Hu et al., 2013). When straw returning to the 15 cm soil layers, the cumulative emission of N_2_O was the largest, which may be due to the fact that the soil layer was close to the ground and the dry-wet alternation was frequent, and the suitable temperature was conducive to the reproduction of microorganisms, which accelerated the decomposition of straw and promoted the emission of N_2_O (Jacinthe and Lal, 2003; Castro et al., 2010). When straw returning to the 15 cm soil layers, the emission of N_2_O was relatively small. On the one hand, it is because deep returning reduced soil bulk density, releases nutrients to the deep layer, and increased NO_3_^–^N, thereby inhibited the activity of denitrifying enzymes. On the other hand, the degree of soil nutrient deficiency in the 40-60 cm soil layer was higher. After straw returning, the fixation of nitrogen by microorganisms was increased, and the concentration of available nitrogen in soil was reduced, thus inhibited the nitrification and denitrification processes and reduced the N_2_O emissions (Gebauer, 2013). Some studies have shown that the emission of CH_4_ in dryland soil was lower, and it is mostly absorbed (Zheng et al., 2021). This may be because the dryland soil was relatively dry, the ventilation condition was good, and oxygen was more likely to diffuse into the soil, so that the CH_4_ was oxidized. It may also be due to the high decomposition rate of organic matter in dryland soil, which is not easy to accumulate organic carbon, thus affecting the production and emission of CH_4_. Therefore, the CH_4_ emissions were not measured in this study.

The cumulative emissions of the soil CO_2_ and N_2_O increased after depth of straw returning treatments, which promoted the GWP of S15, S25 and S40 treatments to be significantly higher than CK treatment. It is worth noting that the GWP was decreased with the increase of straw returning depth. The GHGI is an evaluation index of low-carbon agriculture, which takes into account both the crop yield and global warming potential. In this study, the GHGI was shown a different trend from the GWP. Compared with CK treatments, S25 treatments was no significant difference in 2020, and decreased significantly in 2021 and 2022. This is due to the combined effect of the maize yield and cumulative greenhouse gas emissions, indicating that the appropriate straw returning method can not only be further improved crop yield without the cost of environmental benefits but also improve soil productivity and enhance the carbon sequestration effect of farmland soil, which is an ideal soil improvement and fertilization measure.

## 5 Conclusion

In this study, compared with CK treatments, depth of straw returning were increased the soil SOC and TN, and improved soil quality. The soil quality-related traits were highly correlated with the maize yield, among which S15 and S25 increased yield more obviously, indicating that the improvement of soil quality by depth of straw returning helped to increase maize yield. The analysis of the greenhouse gas emissions showed that the global warming potential gradually decreased with the increase of straw returning depth, and were significantly higher than that of CK treatments. In order to further evaluate the environmental benefits of straw returning, this study measured the GHGI, and the results showed that S25 treatments were decreased significantly compared with CK treatments. These results indicating that the appropriate straw returning depth of can not only be further improved crop yield without the cost of environmental benefits but also improve soil productivity and enhance the carbon sequestration effect of farmland soil, which is an ideal soil improvement and fertilization measure.

## Author's note

We ensure that all maize seeds used in this study originated from Qiqihar Branch of Heilongjiang Academy of Agricultural Sciences in Heilongjiang Province, China. The legality of these seeds complies with the IUCN Policy Statement on Research Involving Species at Risk of Extinction and the Convention on the Trade in Endangered Species of Wild Fauna and Flora. The maize seeds collected in the study are all cultivated maize in China rather than endangered and wild species. These varieties have passed the legal variety certification procedures in China and are licensed for production, planting, and market operations. The authors declare that the cultivation of plants and carrying out study in the Qiqihar maize experiment base of Heilongjiang academy of agricultural sciences complies with all relevant institutional, national and international guidelines and treaties.

## Data availability statement

The original contributions presented in the study are included in the article/supplementary material. Further inquiries can be directed to the corresponding authors.

## Author contributions

JW: Writing – original draft. YH: Data curation, Writing – review & editing. CZ: Formal analysis, Writing – original draft. TX: Investigation, Writing – review & editing. ZQ: Supervision, Writing – original draft. BM: Software, Writing – review & editing. MY: Methodology, Writing – review & editing. LW: Methodology, Writing – original draft. YL: Software, Writing – original draft. QL: Software, Writing – review & editing. XD: Methodology, Writing – review & editing. CQ: Software, Writing – review & editing. BXM: Software, Writing – review & editing.
